# Polystyrene nanoparticles reduce the *Cryptococcus neoformans* virulence via induction of mitochondrial dysfunction

**DOI:** 10.3389/fcimb.2025.1708192

**Published:** 2025-11-05

**Authors:** Dongnan Zheng, Yifan Zhou, Bin Xu, Wenxia Bu, Fengxu Wang, Xinyuan Zhao, Peng Xue, Yuanyuan Ma

**Affiliations:** ^1^ Nantong Key Laboratory of Environmental Toxicology, Institute for Applied Research in Public Health, School of Public Health, Nantong University, Nantong, China; ^2^ Jiangxi Key Laboratory of Oncology(2024SSY06041), JXHC Key Laboratory of Tumor Metastasis, Jiangxi Cancer Hospital, The Second Affiliated Hospital of Nanchang Medical College, Nanchang, China

**Keywords:** *Cryptococcus neoformans*, polystyrene nanoparticles, virulence, capsule, mitochondrial dysfunction

## Abstract

**Introduction:**

*Cryptococcus neoformans* is a fungus that poses a significant threat to human health, with its polysaccharide capsule being a key virulence factor that can upregulate the expression of host gene *ARG1*, encoding arginase-1, which suppresses T-cell-mediated antifungal immune responses. Nanoplastics may cause oxidative and mitochondrial stress in mammalian cells, potentially impacting fungal physiology and pathogenic mechanisms as well.

**Methods:**

We utilized mouse models and fungal burden assays to investigate the effects of polystyrene nanoparticles (PS-NPs) on *C. neoformans* infection. Mice were subjected to oropharyngeal aspiration of 50 μl of 80 nm PS-NPs at a concentration of 5 μg/μl, administered three times a week over a specified duration. To assess the impact of PS-NPs on *C. neoformans* mitochondria, we measured intracellular reactive oxygen species (ROS) levels, mitochondrial superoxide, mitochondrial membrane potential, and intracellular ATP levels in whole fungal cells. Additionally, we performed RNA-Seq analysis and metabolomics studies to evaluate the effects of PS-NPs at a concentration of 0.3 μg/μL on the RNA and metabolic profiles of *C. neoformans* mitochondria.

**Results:**

Our study demonstrated that PS-NPs significantly prolonged the survival of mice infected with *C. neoformans* (*P* = 0.0058). PS-NPs exposure resulted in a 30% reduction in *ARG1* mRNA expression and enhanced T-cell-mediated antifungal immunity. Additionally, PS-NPs inhibited fungal capsule formation by approximately 40% in infected mice and 70% in capsule induction medium. Given the close link between the mitochondria of *C. neoformans* and capsule formation, we further investigated the effects of PS-NPs on mitochondrial function. Exposure to PS-NPs led to mitochondrial dysfunction in *C. neoformans*, as evidenced by a threefold increase in ROS, a 1.7-fold increase in mitochondrial membrane potential, and disruptions in mitochondrial transcription and metabolism.

**Conclusion:**

These results suggest that PS-NPs inhibit the formation of the *C. neoformans* capsule, potentially by inducing mitochondrial dysfunction. Furthermore, the findings highlight the broader implications of PS-NPs on fungal virulence and the dynamics of host-pathogen interactions, underscoring their significance in advancing our understanding of these complex relationships.

## 1 Introduction

*Cryptococcus neoformans* is a significant pathogen capable of causing cryptococcal meningoencephalitis, particularly in individuals with compromised immune systems, such as those with HIV/AIDS. Annually, the disease results in approximately 223,100 deaths within this population (Park et al., 2009; Rajasingham et al., 2017). The World Health Organization recognizes *C. neoformans* as one of four critical priority fungal pathogens (Casalini et al., 2024). Upon inhalation, this fungus primarily infects the lungs before disseminating to the brain, with CD4^+^ T cell-mediated immunity playing a crucial role in controlling the infection. However, *C. neoformans* has a unique ability to suppress immune function, leading to reduced cell-mediated immunity and heightened pro-inflammatory responses (Huffnagle et al., 1991). Within the key virulence factor capsule of *C. neoformans*, the polysaccharide glucuronoxylomannan plays a significant role in triggering the recruitment of neutrophilic myeloid-derived suppressor cells in both mice and patients diagnosed with cryptococcosis (Li et al., 2022). The binding of glucuronoxylomannan to the C-type lectin receptor-2d enhances the immunosuppressive activity of these cells. This binding event initiates the activation of the p38 pathway, leading to the production of arginase-1 (ARG1), which further inhibits T-cell-mediated antifungal responses (Li et al., 2022).

In recent years, environmental concerns about microplastics and nanoplastics have surged due to their pervasive presence and potential detrimental impacts across various ecosystems (Ding et al., 2021; Barguilla et al., 2022; Liu et al., 2022; Li et al., 2023; Tang et al., 2023; Zhai et al., 2024; Bu et al., 2025; Cheng et al., 2025). Emerging evidence suggests that nanoplastics, including polystyrene nanoparticles (PS-NPs), can interact with biological systems, potentially influencing fungal pathogenicity (Ma et al., 2025b). In this study, we examined the effect of PS-NPs on *C. neoformans* infection and found that PS-NPs enhanced survival of *C. neoformans*-infected mice by inhibiting the fungal capsule formation. This study provides new insights into the various impacts of PS-NPs.

## 2 Materials and methods

### 2.1 Strains and media

The H99 strain of *C. neoformans* var. *grubii*, classified as serotype A (MATα), was cultured and maintained on yeast extract peptone dextrose (YPD) medium, which consists of 2% peptone, 1% yeast extract, and 2% dextrose. Additionally, yeast nitrogen base (YNB) medium and capsule induction medium (low-iron medium) were used, prepared according to the methods described in previous studies (Vartivarian et al., 1993; Jung et al., 2008; Saikia et al., 2014).

### 2.2 Mouse models and fungal burden assay

Male BALB/c mice (6–7 weeks old) were obtained from the Experimental Animal Center at Nantong University (Nantong, China) for virulence assays. Wild-type (WT) *C. neoformans* cells were cultured overnight in YPD medium at 30°C under shaking (130 rpm), washed with phosphate buffered saline (PBS), and resuspended at a concentration of 1×10 cells ml^-1^ in PBS. Inoculation was performed by intranasal instillation of 50 μl of the cell suspension, corresponding to an inoculum of 5×10^2^ cells per mouse. The first group of 11 mice infected with the WT strain received 50 μl of 5 μg/μl PS-NPs (80 nm) treatment via oropharyngeal aspiration three times a week (Tuesday, Thursday, and Saturday). The second group of 11 infected mice were treated with PBS. The other two groups, without infection, received only PBS or PS-NPs. Mice were monitored daily for health status, and those reaching the humane endpoint were euthanized using CO_2_ asphyxiation. After 28 days post-inoculation, infected mice were euthanized using CO_2_ inhalation to evaluate fungal loads in organs. Their organs were removed, weighed, and homogenized in 1 ml of PBS with a MixerMill (Retsch). Serial dilutions of the homogenates were plated on YPD agar plates containing 35 μg/ml chloramphenicol, and the number of colony-forming units (CFUs) was determined after 48 hours of incubation at 30°C. Ethical guidelines regarding animal use set by Nantong University were strictly adhered to, ensuring minimal suffering for all mice.

### 2.3 Capsule analysis

To analyze the capsule of *C. neoformans* cells in the lungs, lung tissues were fixed in 4% paraformaldehyde, embedded in paraffin, and sectioned into 3 μm slices. Hematoxylin and eosin (HE) staining was performed, and the sections were examined under Nikon ECLIPSE Ti2 microscopy. For capsule analysis of *C. neoformans* in the capsule induction medium, the cells were cultured in the medium for 18 h with or without PS-NPs (0.3 μg/μl) at 30 °C under shaking at 130 rpm. Subsequently, measurements were taken for cell diameter, and capsule size of cells staining with India ink. A total of 50 cells were analyzed per condition, and the data were quantified using ImageJ software. Statistical analyses were performed to assess differences between groups. Data are presented as mean ± standard deviation (SD). To determine statistical significance, a t-test (two-tailed) was utilized.

### 2.4 Measurement of intracellular ROS levels, mitochondrial superoxide and mitochondrial membrane potential and intracellular ATP level

*C. neoformans* cells were cultured overnight at 30°C with shaking at 130 rpm in YPD medium. The cells were then washed twice with PBS and inoculated at an OD_600_ of 0.8 in capsule induction medium, with or without 80 nm non-fluorescent PS-NPs (0.3 μg/μl), and incubated for 18 hours at 30 °C with shaking at 130 rpm. For the assessment of intracellular ROS and mitochondrial function, cells were subsequently stained for various assays. To detect intracellular ROS, cells were incubated with 2',7'-Dichlorofluorescein Diacetate (DCFH-DA, 16 μM) at 30°C for 1 hour. Mitochondrial superoxide levels were measured by treating the cells with Mito-SOX Red (50 mM) under the same conditions. For assessing mitochondrial membrane potential, cells were stained with Mito-Tracker Red CMXRos (Mito-Tracker, 50 nM) for 1 hour at 30°C. The fluorescence intensity of the stained cells (5×10^7^) was measured using a multimode microplate reader (TECAN Infinite E Plex). Intracellular ATP levels were quantified using the BacTiter-Glo Microbial Cell Viability Assay kit (Promega, USA), with signal detection performed on the same microplate reader (TECAN Infinite E Plex). Fluorescence data were normalized to the total cell number to ensure comparability across samples. The experiment was conducted in triplicate.

### 2.5 RNA-seq analysis

To investigate the effects of PS-NPs on the transcriptome of *C. neoformans*, WT cells were cultivated in YPD medium at 30°C for 16 hours. After washing, the cells were adjusted to a concentration of 4.0 × 10^7^ cells/mL in YNB medium, with or without the addition of 80 nm non-fluorescent PS-NPs at 0.3 μg/μL. Following an 18-hour incubation, the cells were harvested, washed, and flash-frozen in liquid nitrogen. Total RNA was extracted from the cell pellets using TRIzol. RNA concentration and integrity were assessed using standard methods. Indexed RNA-Seq libraries were generated from 800 ng of total RNA and sequenced on an Illumina HiSeq2500 platform. Read alignment was conducted using Bowtie, and transcript abundances were determined via the RNA-Seq by expectation-maximization tool. Differentially expressed genes (DEGs) were identified with a false discovery rate (FDR) < 0.05 and fold change (FC) ≥ 2. Expression data visualization, including volcano plots, was performed using R (version 4.2.2) and the ggplot2 package. Gene Ontology (GO) analysis was conducted for DEGs using the GO database (Ashburner et al., 2000), and significantly enriched terms were identified using a hypergeometric test. KEGG pathway analysis was used for functional annotation (Kanehisa and Goto, 2000). Validation of RNA-Seq results was performed with quantitative PCR (qPCR) using specific primers (see **Supplementary Table S1** for details).

### 2.6 Metabolomics studies

To evaluate the impact of PS-NPs on metabolomics, we cultured three replicate cultures of *C. neoformans* WT cells in YPD medium for 16 hours at 30 °C. After washing, cells were adjusted to a specific concentration in YNB medium, either with or without PS-NPs, and incubated for an additional 18 hours at 30°C. Following harvest and washing, the cells were frozen in liquid nitrogen. The freeze-dried pellets (~80 mg) were pulverized, and 1000 μL of a methanol/acetonitrile/water (2:2:1, v/v/v) solution was added for metabolite extraction. After centrifugation at 14,000 g and 4 °C for 15 minutes, the supernatant was evaporated, and the samples were reconstituted in 100 μL of acetonitrile/water (1:1, v/v) for LC-MS analysis. We used a UHPLC system coupled with a quadrupole time-of-flight mass spectrometer to perform HILIC separation. The mobile phase consisted of ammonium acetate and ammonium hydroxide in water, and acetonitrile, with a gradient profile tailored for optimal metabolite resolution. Mass spectrometry parameters were set for both positive and negative ion modes, scanning m/z ranges of 60–1000 Da and 25–1000 Da, respectively. Quality control (QC) samples were included to monitor systematic errors during extraction and analysis (Dunn et al., 2011). Metabolite data were normalized based on total peak area to ensure consistency across samples. Principal Component Analysis (PCA) was performed using the gmodels package in R (Warnes et al., 2015), and metabolites with a p-value of <0.05 from differential analysis were identified. The KEGG database was utilized for pathway enrichment analysis, with FDR correction (threshold set at FDR ≤ 0.05) applied to determine significantly enriched pathways associated with the differential metabolites (Kanehisa and Goto, 2000).

## 3 Results

### 3.1 PS-NPs exposure mitigates *C. neoformans* infection

**Figure 1:**
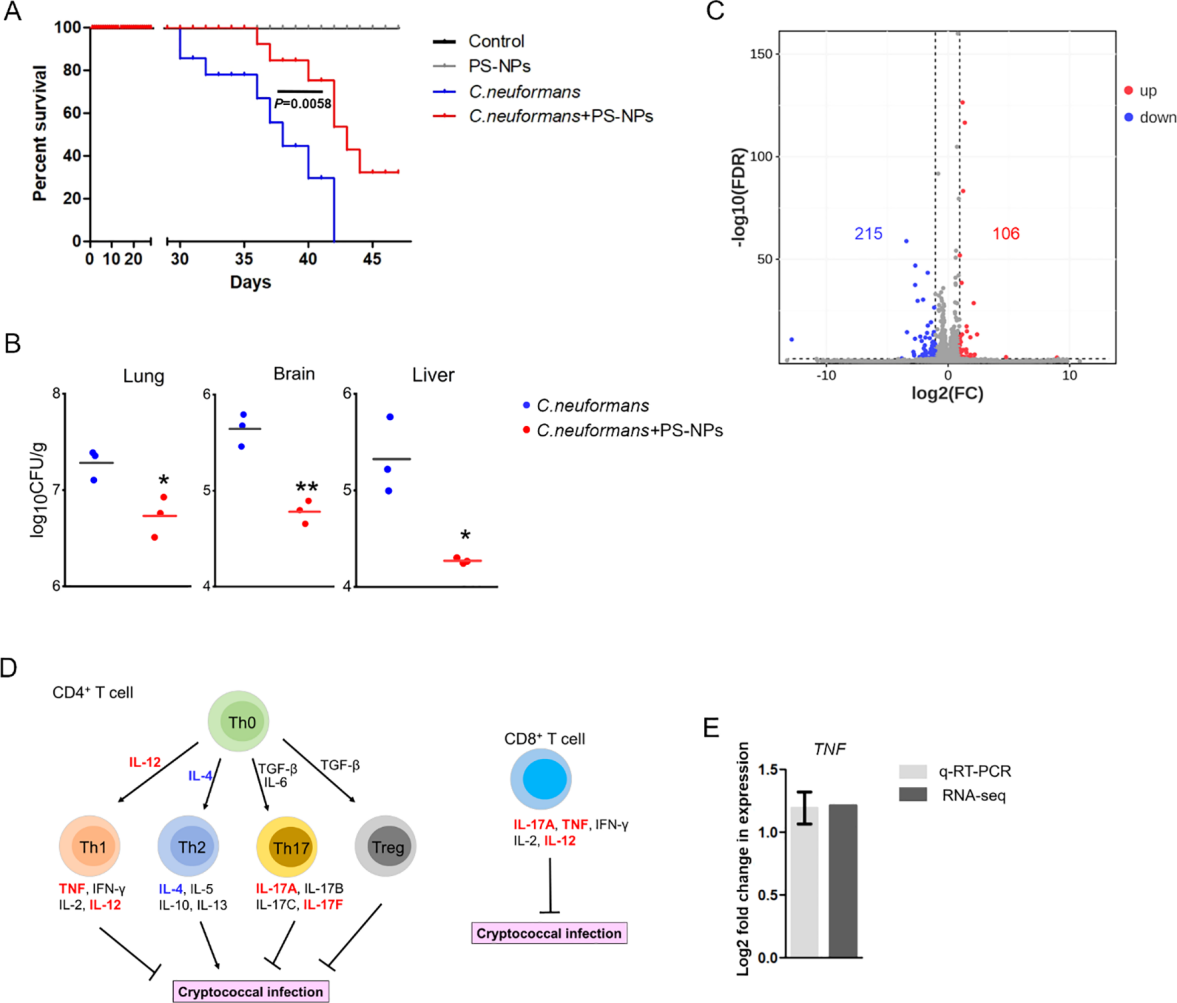
PS-NPs attenuate *C*. *neoformans* virulence in the mice model of inhalation exposure. **(A)** Survival curve. Male BALB/c mice were intranasally infected with 5×10² cells of the WT strain (H99), then divided into two groups after one day. One group received 50 μl of 0.08 μm PS-NPs at a concentration of 5 μg/μl three times a week; the other group received PBS. Survival differences were analyzed using the log rank Mantel-Cox test. **(B)** Fungal burden. Fungal cells in the lungs, brains, and livers of infected mice were assessed after 28 days. Significant differences were observed between the two treatment groups as determined by the Mann-Whitney U test (*, *P*<0.05; **, *P*<0.01). **(C)** Gene regulation. Analysis revealed 215 downregulated (green) and 106 upregulated (red) genes in the lungs of infected mice treated with or without PS-NPs after 28 days. **(D)** Cytokine expression. PS-NPs regulated T-cell cytokine gene expression, highlighted by upregulated genes in red and downregulated genes in blue. **(E)** *TNF* gene expression. Transcript levels of the *TNF* gene were compared via RNA-Seq and qRT-PCR, with a significance threshold of *P*<0.05 and fold change cut-off ≥ 2.

**Figure 2:**
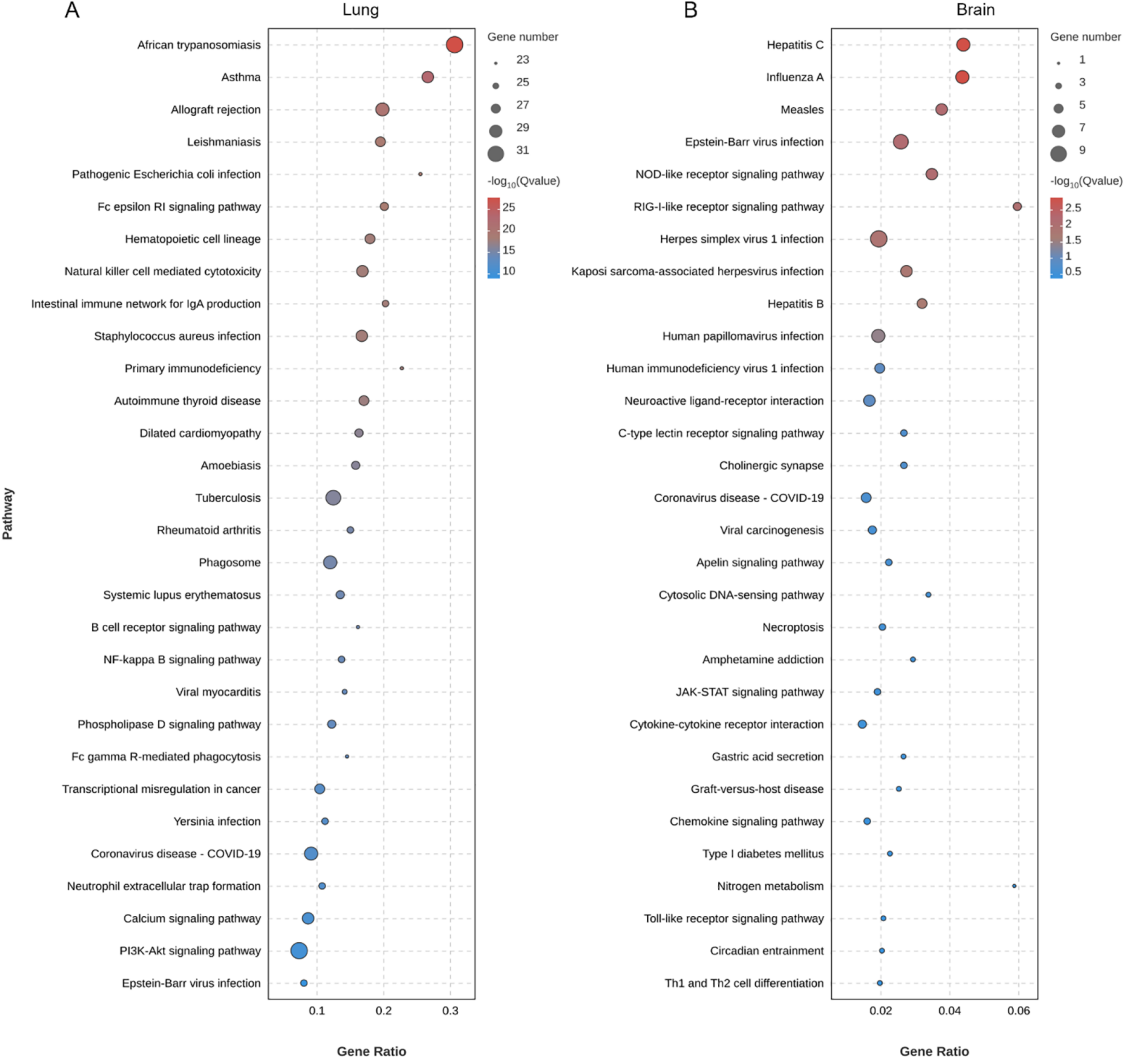
Enrichment analysis of KEGG pathways in **(A)** lung and **(B)** brain of *C*. *neoformans*-infected mice treated with PS-NPs. The top 30 enriched KEGG pathways identified through DEGs. Statistical significance for these pathways was assessed using p-values corrected for false discovery rate (FDR), with an FDR threshold set at ≤ 0.05.

Exposure to 80 nm diameter PS-NPs significantly prolonged the survival of mice infected with the *C. neoformans* strain H99, with a survival analysis indicating a statistically significant improvement (*P* = 0.0058) (**Figure 1A**). Furthermore, treatment with PS-NPs led to a notable reduction in fungal burden in the lung, brain, and liver tissues of infected mice, with statistical analysis showing *P* < 0.05 across these organs (**Figure 1B**). These findings imply that PS-NPs can effectively mitigate *C. neoformans* infection. To understand the underlying immune mechanisms, we explored the influence of PS-NPs on T cell-mediated immune responses against *C. neoformans*. RNA-Seq analysis comparing the transcript profiles of mouse lung tissue from PS-NPs-exposed and non-exposed mice revealed changes in the expression of 321 genes, with 106 genes being upregulated and 215 downregulated (**Figure 1C**). We focused on the transcript levels of T-cell cytokines known to play critical roles in the host defense against cryptococcal infections. Notably, transcripts for cytokines TNF, IL-12, IL12A, and IL17F were upregulated in response to PS-NPs exposure, indicating a potential enhancement of protective immune responses (**Figures 1D, E**). In contrast, levels of IL-4 and IL-10, which are associated with immunosuppression and exacerbation of cryptococcal diseases, were downregulated following PS-NPs treatment (**Figure 1D**). Moreover, KEGG pathway analysis revealed an enrichment of immune system-associated terms within the lung tissue of PS-NPs exposed infected mice, particularly highlighting the NF-kappa B signaling pathway (**Figure 2A**). In the brain of these PS-NPs exposed mice, we observed alterations in immune responses, especially regarding the differentiation of Th1 and Th2 cells (**Figure 2B**). Overall, PS-NPs exposure appears to significantly enhance T-cell mediated antifungal immunity in mice infected with *C. neoformans*.

### 3.2 PS-NPs exposure suppresses *C. neoformans* capsule formation

**Figure 3:**
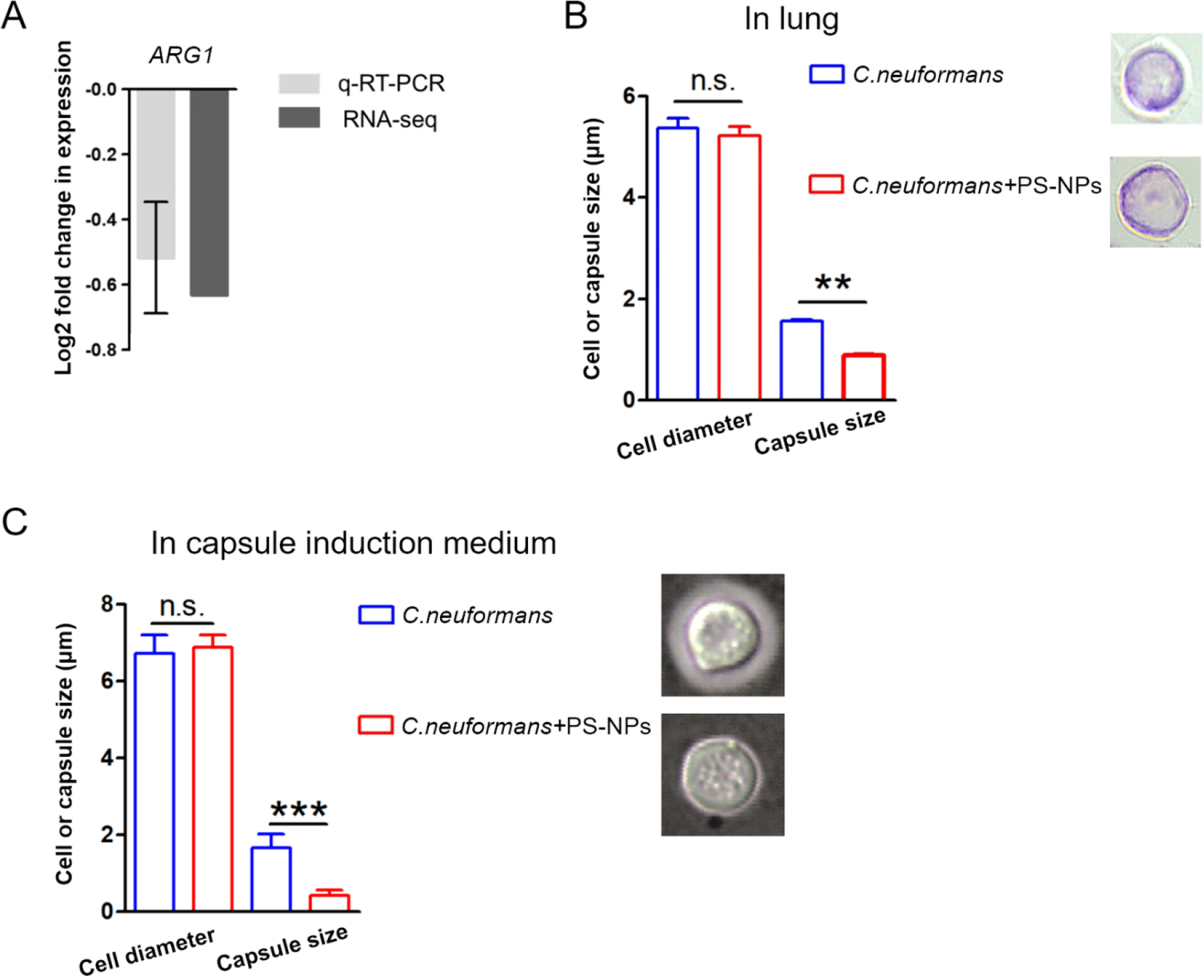
PS-NPs inhibit capsule formation. **(A)** *ARG1* gene transcript levels were analyzed by RNA-Seq and qRT-PCR. **(B)** Cell diameter and capsule size of fifty *C*. *neoformans* cells from infected mouse lungs were measured after 28 days of treatment with or without PS-NPs. **(C)** Cell diameter and capsule size were also measured for fifty *C*. *neoformans* cells cultured in capsule induction medium for 18 hours, with or without PS-NPs. Capsule formation was assessed using India ink staining. Statistical analysis via Student’s t-test showed significant differences in capsule size, marked by ** (*P*<0.01) and *** (*P*<0.001). n.s., not statistically significant.

The capsule of *C. neoformans* induces the expression of *ARG1* to inhibit T-cell mediated antifungal immunity (Li et al., 2022). Our findings demonstrated a decrease in *ARG1* mRNA expression following exposure to PS-NPs (**Figure 3A**). Subsequently, the size of the *C. neoformans* capsule was examined in mice and capsule induction medium with or without PS-NPs treatment. It was found that PS-NPs exposure suppressed the formation of the *C. neoformans* capsule in both mice lungs (**Figure 3B**) and capsule induction medium (**Figure 3C**). These results indicate that PS-NPs exposure leads to reduced capsule formation in *C. neoformans*.

### 3.3 PS-NPs induce *C. neoformans* mitochondrial dysfunction

**Figure 4:**
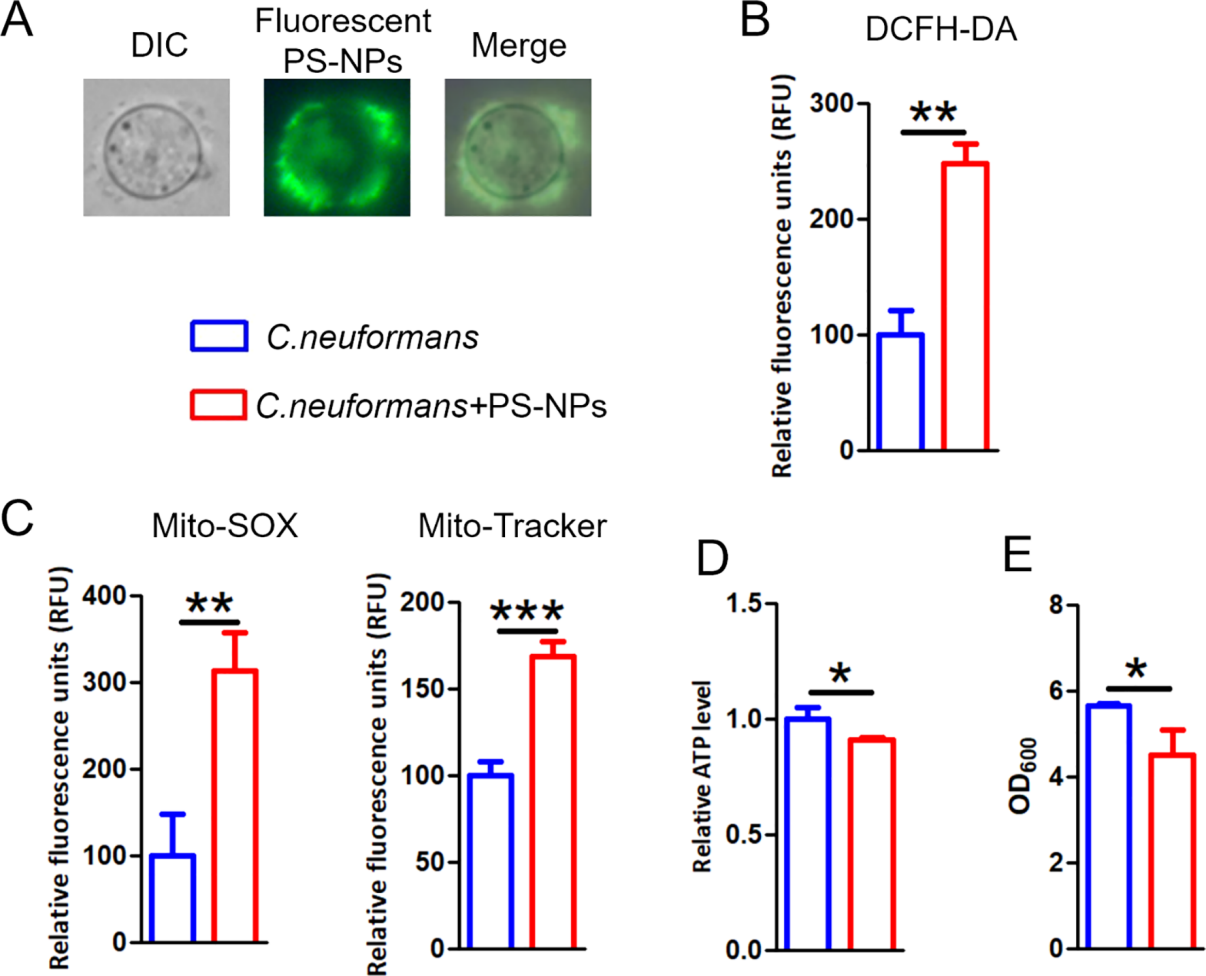
PS-NPs provoke mitochondria damage. **(A)** Images of *C*. *neoformans* cultured with 80 nm green fluorescent PS-NPs. **(B)** ROS accumulation assessed by DCFH-DA staining following exposure to non-fluorescent PS-NPs. **(C)** Mitochondrial membrane potential and superoxide levels evaluated using Mito-Tracker Red CMXRos and Mito-SOX Red, respectively, after PS-NPs treatment. **(D)** Measurement of intracellular ATP levels after PS-NPs exposure. **(E)** Cell densities (OD_600_) measured after treatment. * (P<0.05), ** (P<0.01) and *** (P<0.001).

We first investigated the impact of PS-NPs on ROS levels and mitochondrial function in *C. neoformans*. PS-NPs adhered to the cell wall and entered the cells (**Figure 4A**). Treatment with PS-NPs led to a significant increase in intracellular ROS levels, as shown by higher DCFH-DA staining compared to untreated cells (**Figure 4B**). Additionally, we observed that PS-NPs treatment resulted in increased mitochondrial superoxide and membrane potential in *C. neoformans* (**Figure 4C**). However, this treatment also reduced intracellular ATP levels (**Figure 4D**) and inhibited fungal cell growth (**Figure 4E**).

**Figure 5:**
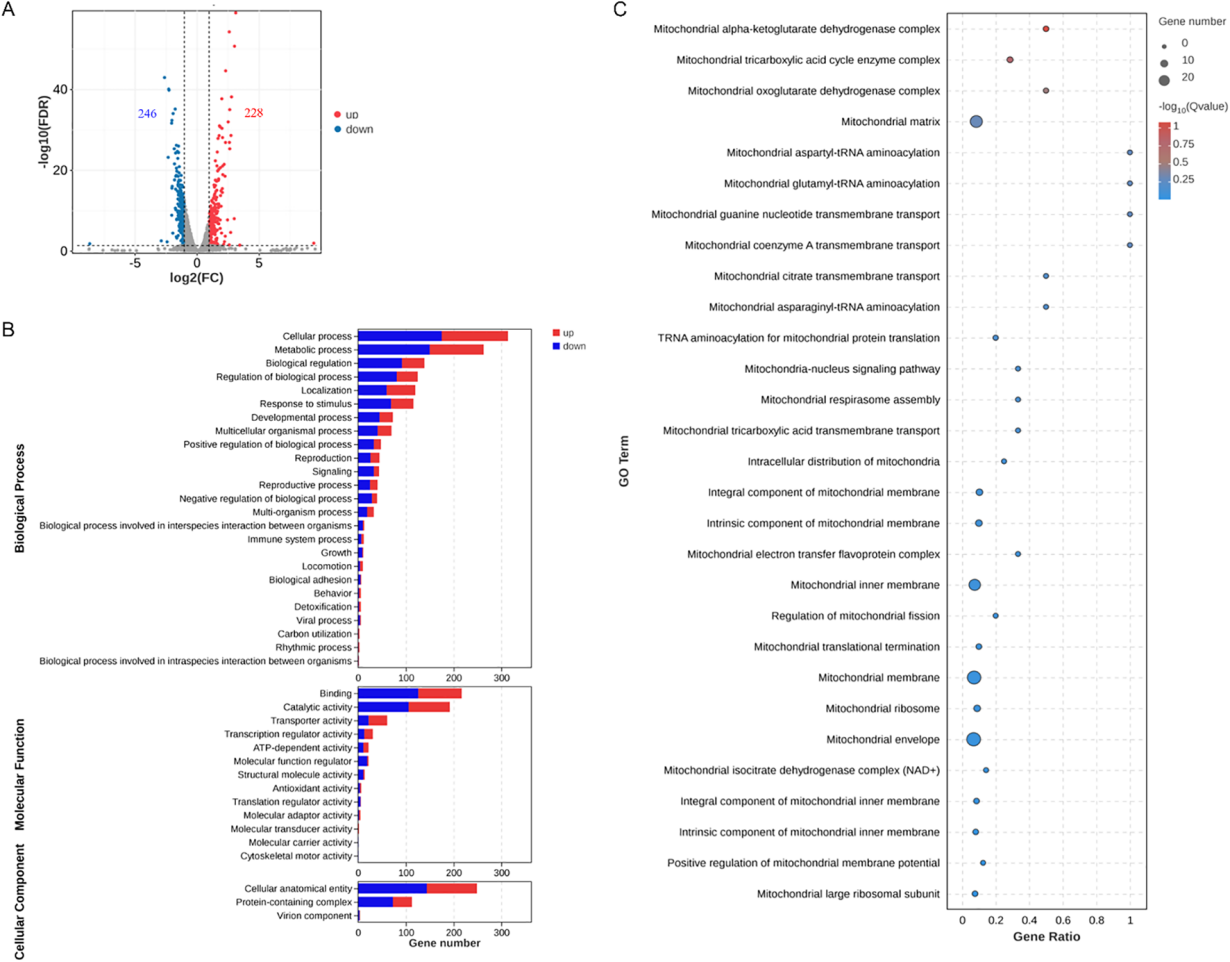
PS-NPs modulate the transcriptional regulation of genes associated with *C*. *neoformans* mitochondrial functions. **(A)** A total of 246 genes were downregulated (green) and 228 genes were upregulated (red) in *C*. *neoformans* after treatment with PS-NPs. Differential expression was defined at FDR < 0.05 and fold change ≥ 2. **(B)** GO categories for the differentially expressed genes identified via RNA-seq analysis of *C*. *neoformans* cells treated with PS-NPs. **(C)** Gene Set Enrichment Analysis (GSEA) shows enrichment for the top 30 pathways directly related to mitochondrial function. Improved GO and GSEA pathways highlight disruption of mitochondrial energy and antioxidant functions.

**Figure 6:**
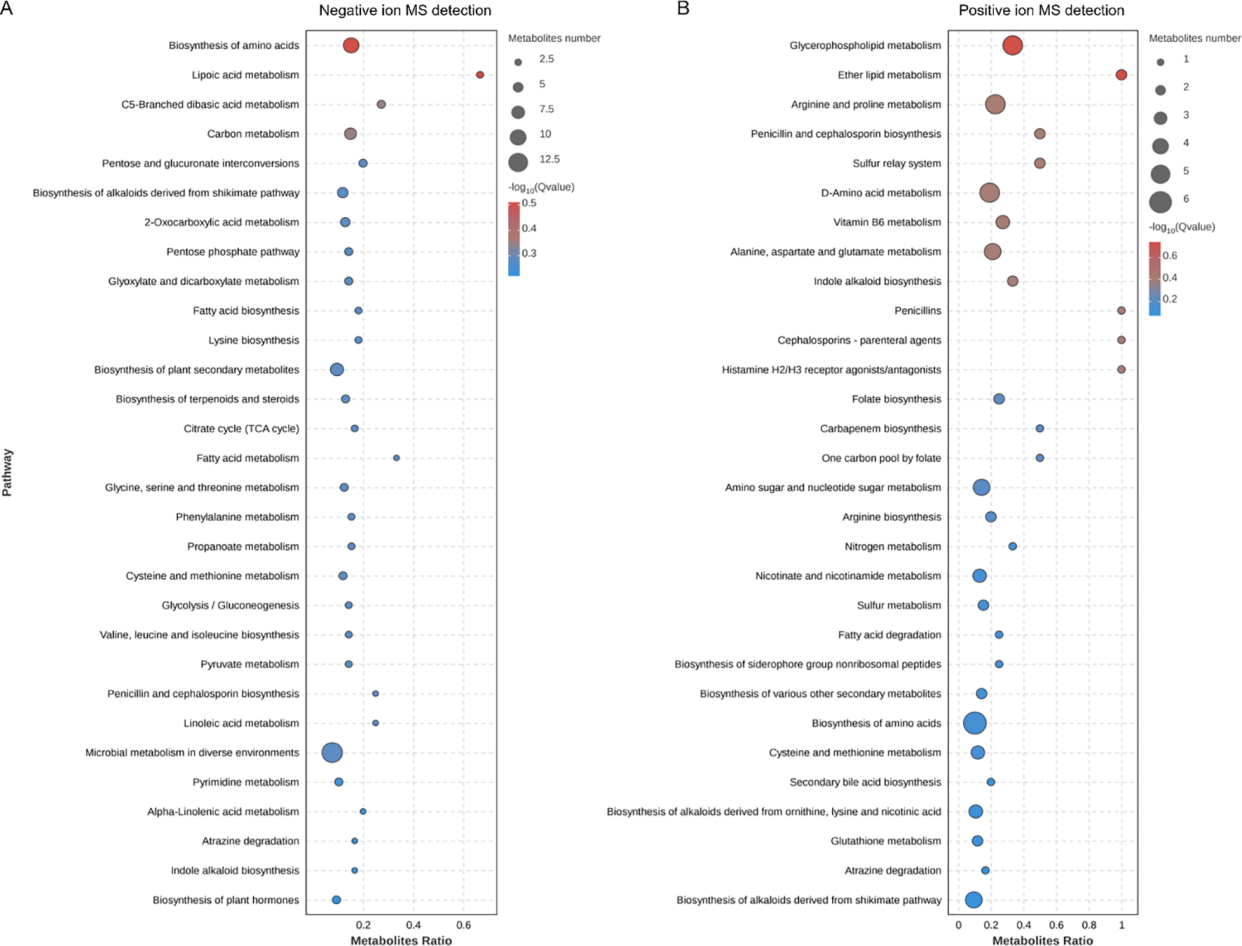
KEGG pathway enrichment analysis in PS-NPs-treated *C*. *neoformans*. **(A)** Negative ion MS detection. **(B)** Positive ion MS detection. Top 30 enriched pathways based on differentially expressed metabolites, identified using a significance threshold of *P* < 0.05, highlighting key metabolic alterations associated with the treatment.

We further explored the effects of PS-NPs on mitochondrial transcription and metabolism in *C. neoformans*. Our transcriptomic analysis revealed differential expression levels for 474 genes, with 228 genes upregulated and 246 genes downregulated (**Figure 5A**). GO analysis showed disturbances in molecular function categories related to ATP-dependent and antioxidant activity (**Figure 5B**). We noted that PS-NPs interfered with mitochondrial transcription, which affected various components, including mitochondrial tricarboxylic acid (TCA) cycle enzyme complexes and the assembly of the mitochondrial respirasome (**Figure 5C**). Moreover, metabolomics analysis indicated that PS-NPs disrupted mitochondrial metabolic pathways, leading to significant alterations in the TCA cycle and glutathione (GSH) metabolism. Specifically, negative ion MS revealed changes in the TCA cycle (**Figure 6A**), while positive ion MS showed alterations in GSH metabolism (**Figure 6B**). Overall, these results indicate that PS-NPs exposure altered mitochondrial activity, reduced ATP levels, and disrupted TCA and GSH pathways.

## 4 Discussion

The environmental and toxicological impacts of nanoplastics and microplastics have become significant areas of concern globally, particularly regarding their potential threats to ecosystems and human health (Sana et al., 2020; Mitrano et al., 2021; Aeschlimann et al., 2022; Abdolahpur Monikh et al., 2023; Marfella et al., 2024). A growing body of research indicates that oxidative stress and inflammation are common mechanisms associated with various environmental pollutants (Zheng et al., 2021; Liu et al., 2024), which also apply to exposures from nanoplastics and microplastics. The biological effects of these pollutants include oxidative stress, inflammatory responses, and genetic toxicity, reflecting their profound impact on living organisms and the environment (Yin et al., 2021; Babaei et al., 2022; Ding et al., 2023; Tang et al., 2023).

**Figure 7:**
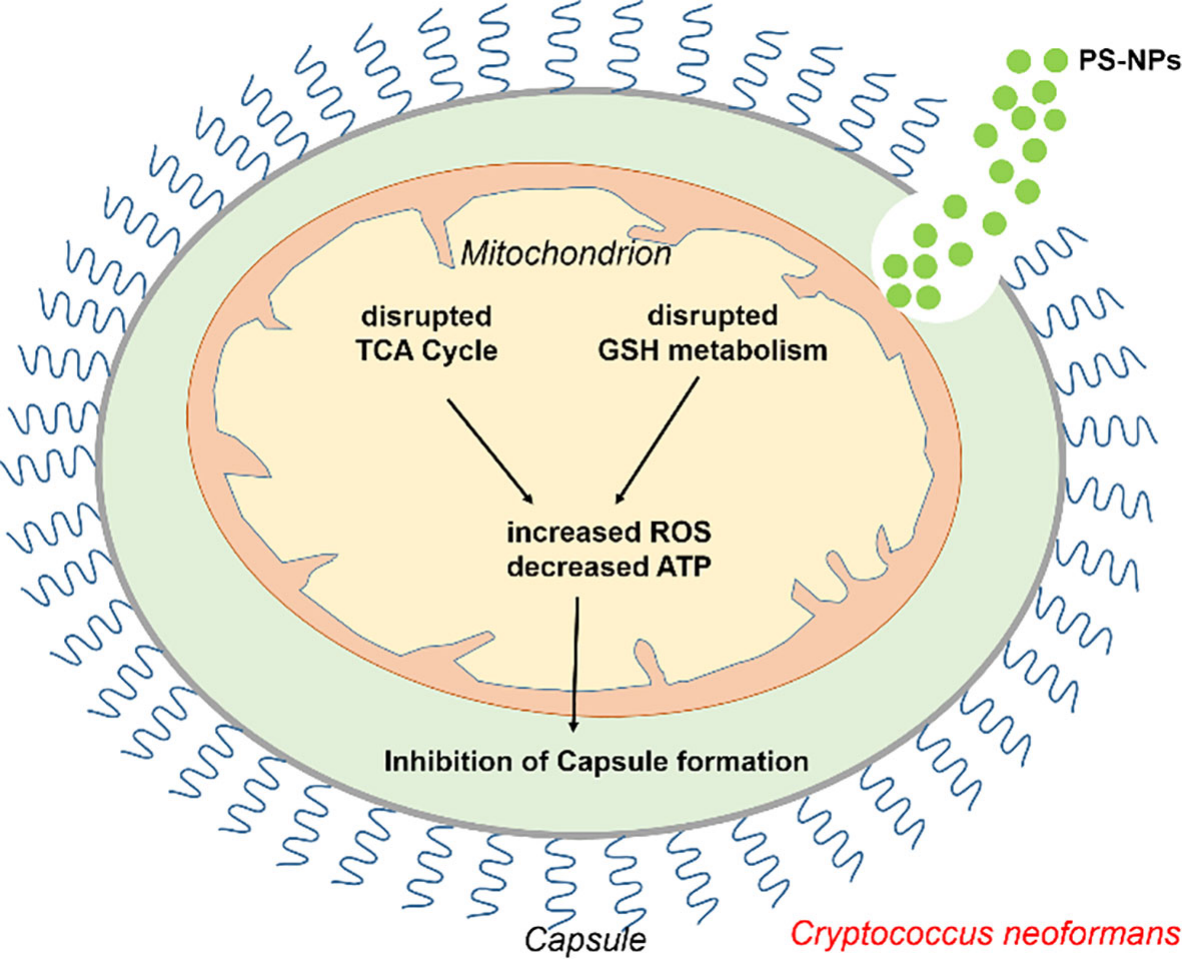
Schematic representation of how PS-NPs modulate *C. neoformans* virulence by inducing mitochondrial dysfunction. PS-NPs induce mitochondrial dysfunction by increasing ROS production and depleting ATP, ultimately inhibiting capsule formation.

This study presents a novel finding: PS-NPs reduce the virulence of *C. neoformans*, potentially via mitochondrial disruption. The dysfunction induced by PS-NPs significantly impacts the metabolic pathways and pathogenicity of *C. neoformans* (**Figure 7**). Such mitochondrial disruption not only impairs normal physiological processes but may also open new avenues for enhancing antifungal resistance (Ma et al., 2025b). While it is apparent that oxidative damage could drive *C. neoformans* to develop increased resistance to antifungal treatments via genetic mutations or adaptive changes, we must avoid over-speculation in this regard until further data is available. It is important to note that the nanoplastics used in this study were purchased from a commercial supplier, and their shapes may differ from those found in the environment. Additionally, the concentrations of nanoplastics employed in our laboratory setting may vary from those encountered in natural environments. These factors may influence the interactions between nanoplastics and *C. neoformans*, somewhat limiting the generalizability of our findings.

The role of mitochondria in cellular energy metabolism and stress response reinforces their significance in developing potential antifungal therapies. Mitochondria are integral to ATP synthesis and critically influence the activity and expression of drug efflux pumps (Calderone et al., 2015; Black et al., 2021; Xue et al., 2024; Ma et al., 2025a, 2025). Given the emerging focus on mitochondria as targets for novel drugs, understanding the molecular mechanisms behind PS-NP-induced mitochondrial dysfunction could be crucial for devising effective antifungal treatment strategies. On an ecological scale, the long-term persistence and toxicity of PS-NPs require thorough investigation. Their accumulation in natural environments could disrupt fungal community structures and dynamics, potentially favoring certain species while inhibiting others. Such changes may undermine ecosystem stability and diversity, necessitating future studies to assess the long-term effects of PS-NPs on ecological health.

In summary, the interactions between PS-NPs and *C. neoformans* shed light on the role of nanoplastics in modulating fungal virulence. They also offer insights into environmental factors contributing to fungal resistance, emphasizing the importance of systematic analyses of the environmental and toxicological impacts of PS-NPs. This understanding is paramount for informing public health risk management and guiding future environmental protection strategies.

## Data Availability

The RNA-Seq data can be accessed in the Genome Sequence Archive (GSA) under the identifiers CRA029802 and CRA029810 at https://ngdc.cncb.ac.cn/gsa. Additionally, the metabolomics data is available in the OMIX database (OMIX011864) at this link: https://ngdc.cncb.ac.cn/omix.

## Supplementary material

Supplementary Table 1 Primer sequences used for qRT-PCR. F: forward primer; R: reverse primer.

## References

[B1] Abdolahpur Monikh F. Baun A. Hartmann N. B. Kortet R. Akkanen J. Lee J. S.. (2023). Exposure protocol for ecotoxicity testing of microplastics and nanoplastics. Nat. Protoc. 18, 3534–3564. doi: 10.1038/s41596-023-00886-9, PMID: 37816903

[B2] Aeschlimann M. Li G. Kanji Z. A. Mitrano D. M. (2022). Potential impacts of atmospheric microplastics and nanoplastics on cloud formation processes. Nat. Geosci. 15, 967–975. doi: 10.1038/s41561-022-01051-9, PMID: 36532143 PMC7613933

[B3] Ashburner M. Ball C. A. Blake J. A. Botstein D. Butler H. Cherry J. M.. (2000). Gene ontology: tool for the unification of biology. Nat. Genet. 25, 25–29. doi: 10.1038/75556, PMID: 10802651 PMC3037419

[B4] Babaei A. A. Rafiee M. Khodagholi F. Ahmadpour E. Amereh F. (2022). Nanoplastics-induced oxidative stress, antioxidant defense, and physiological response in exposed Wistar albino rats. Environ. Sci pollut. Res. 29 (8), 11332–11344. doi: 10.1007/s11356-021-15920-0, PMID: 34535860

[B5] Barguilla I. Domenech J. Ballesteros S. Rubio L. Marcos R. Hernández A. (2022). Long-term exposure to nanoplastics alters molecular and functional traits related to the carcinogenic process. J. Hazardous Materials 438, 129470. doi: 10.1016/j.jhazmat.2022.129470, PMID: 35785738

[B6] Black B. Lee C. Horianopoulos L. C. Jung W. H. Kronstad J. W. (2021). Respiring to infect: Emerging links between mitochondria, the electron transport chain, and fungal pathogenesis. PloS Pathog. 17, e1009661. doi: 10.1371/journal.ppat.1009661, PMID: 34237096 PMC8266039

[B7] Bu W. Yu M. Ma X. Shen Z. Ruan J. Qu Y.. (2025). Gender-specific effects of prenatal polystyrene nanoparticle exposure on offspring lung development. Toxicol. Lett. 407, 1–16. doi: 10.1016/j.toxlet.2025.03.001, PMID: 40088994

[B8] Calderone R. Li D. Traven A. (2015). System-level impact of mitochondria on fungal virulence: to metabolism and beyond. FEMS yeast Res. 15, fov027. doi: 10.1093/femsyr/fov027, PMID: 26002841 PMC4542695

[B9] Casalini G. Giacomelli A. Antinori S. (2024). The WHO fungal priority pathogens list: a crucial reappraisal to review the prioritisation. Lancet Microbe 5, 717–724. doi: 10.1016/S2666-5247(24)00042-9, PMID: 38608682

[B10] Cheng D. Zheng D. Jiang M. Jin Y. Liu R. Zhou Y.. (2025). Inhibition of iron ion accumulation alleviates polystyrene nanoplastics-induced pulmonary fibroblast proliferation and activation. Int. Immunopharmacol 164, 115367. doi: 10.1016/j.intimp.2025.115367, PMID: 40811949

[B11] Ding R. Ma Y. Li T. Sun M. Sun Z. Duan J. (2023). The detrimental effects of micro-and nano-plastics on digestive system: An overview of oxidative stress-related adverse outcome pathway. Sci Total Environ. 878, 163144. doi: 10.1016/j.scitotenv.2023.163144, PMID: 37003332

[B12] Ding Y. Zhang R. Li B. Du Y. Li J. Tong X.. (2021). Tissue distribution of polystyrene nanoplastics in mice and their entry, transport, and cytotoxicity to GES-1 cells. Environ. pollut. 280, 116974. doi: 10.1016/j.envpol.2021.116974, PMID: 33784569

[B13] Dunn W. B. Broadhurst D. Begley P. Zelena E. Francis-McIntyre S. Anderson N.. (2011). Procedures for large-scale metabolic profiling of serum and plasma using gas chromatography and liquid chromatography coupled to mass spectrometry. Nat. Protoc. 6, 1060–1083. doi: 10.1038/nprot.2011.335, PMID: 21720319

[B14] Huffnagle G. B. Yates J. Lipscomb M. F. (1991). Immunity to a pulmonary Cryptococcus neoformans infection requires both CD4+ and CD8+ T cells. J. Exp. Med. 173, 793–800. doi: 10.1084/jem.173.4.793, PMID: 1672543 PMC2190813

[B15] Jung W. H. Sham A. Lian T. Singh A. Kosman D. J. Kronstad J. W. (2008). Iron source preference and regulation of iron uptake in Cryptococcus neoformans. PloS Pathog. 4, e45. doi: 10.1371/journal.ppat.0040045, PMID: 18282105 PMC2242830

[B16] Kanehisa M. Goto S. (2000). KEGG: kyoto encyclopedia of genes and genomes. Nucleic Acids Res. 28, 27–30. doi: 10.1093/nar/28.1.27, PMID: 10592173 PMC102409

[B17] Li Y. N. Wang Z. W. Li F. Zhou L. H. Jiang Y. S. Yu Y.. (2022). Inhibition of myeloid-derived suppressor cell arginase-1 production enhances T-cell-based immunotherapy against *Cryptococcus neoformans* infection. Nat. Commun. 13, 4074. doi: 10.1038/s41467-022-31723-4, PMID: 35835754 PMC9283461

[B18] Li Z. Xu T. Peng L. Tang X. Chi Q. Li M.. (2023). Polystyrene nanoplastics aggravates lipopolysaccharide-induced apoptosis in mouse kidney cells by regulating IRE1/XBP1 endoplasmic reticulum stress pathway *via* oxidative stress. J. Cell. Physiol. 238, 151–164. doi: 10.1002/jcp.30913, PMID: 36370432

[B19] Liu L. Luo C. Zheng D. Wang X. Wang R. Ding W.. (2024). TRPML1 contributes to antimony-induced nephrotoxicity by initiating ferroptosis *via* chaperone-mediated autophagy. Food Chem. Toxicol. 184, 114378. doi: 10.1016/j.fct.2023.114378, PMID: 38097005

[B20] Liu X. Zhao Y. Dou J. Hou Q. Cheng J. Jiang X. (2022). Bioeffects of inhaled nanoplastics on neurons and alteration of animal behaviors through deposition in the brain. Nano Lett. 22, 1091–1099. doi: 10.1021/acs.nanolett.1c04184, PMID: 35089039

[B21] Ma Y. Zhou Y. Jia T. Zhuang Z. Xue P. Yang L. (2025a). Deciphering the role of mitochondria in human fungal drug resistance. Mycology, 1–14. doi: 10.1080/21501203.2025.2473507 40083404

[B22] Ma Y. Zhou Y. Zheng D. Bu W. Wang F. Zhao X.. (2025b). Nanoplastics and fungi: exploring dual roles in degradation and pathogenicity. Front. Microbiol. Volume 16. doi: 10.3389/fmicb.2025.1679160, PMID: 41132370 PMC12540466

[B23] Marfella R. Prattichizzo F. Sardu C. Fulgenzi G. Graciotti L. Spadoni T.. (2024). Microplastics and nanoplastics in atheromas and cardiovascular events. New Engl. J. Med. 390, 900–910. doi: 10.1056/NEJMoa2309822, PMID: 38446676 PMC11009876

[B24] Mitrano D. M. Wick P. Nowack B. (2021). Placing nanoplastics in the context of global plastic pollution. Nat. Nanotechnology 16, 491–500. doi: 10.1038/s41565-021-00888-2, PMID: 33927363

[B25] Park B. J. Wannemuehler K. A. Marston B. J. Govender N. Pappas P. G. Chiller T. M. (2009). Estimation of the current global burden of cryptococcal meningitis among persons living with HIV/AIDS. Aids 23, 525–530. doi: 10.1097/QAD.0b013e328322ffac, PMID: 19182676

[B26] Rajasingham R. Smith R. M. Park B. J. Jarvis J. N. Govender N. P. Chiller T. M.. (2017). Global burden of disease of HIV-associated cryptococcal meningitis: an updated analysis. Lancet Infect. Dis. 17, 873–881. doi: 10.1016/S1473-3099(17)30243-8, PMID: 28483415 PMC5818156

[B27] Saikia S. Oliveira D. Hu G. Kronstad J. (2014). Role of ferric reductases in iron acquisition and virulence in the fungal pathogen *Cryptococcus neoformans* . Infection Immun. 82, 839–850. doi: 10.1128/IAI.01357-13, PMID: 24478097 PMC3911385

[B28] Sana S. S. Dogiparthi L. K. Gangadhar L. Chakravorty A. Abhishek N. (2020). Effects of microplastics and nanoplastics on marine environment and human health. Environ. Sci pollut. Res. 27, 44743–44756. doi: 10.1007/s11356-020-10573-x, PMID: 32876819

[B29] Tang J. Bu W. Hu W. Zhao Z. Liu L. Luo C.. (2023). Ferroptosis is involved in sex-specific small intestinal toxicity in the offspring of adult mice exposed to polystyrene nanoplastics during pregnancy. ACS nano 17, 2440–2449. doi: 10.1021/acsnano.2c09729, PMID: 36728677

[B30] Vartivarian S. E. Anaissie E. J. Cowart R. E. Sprigg H. A. Tingler M. J. Jacobson E. S. (1993). Regulation of cryptococcal capsular polysaccharide by iron. J. Infect. Dis. 167, 186–190. doi: 10.1093/infdis/167.1.186, PMID: 8418165

[B31] Warnes G. R. Bolker B. Lumley T. Johnson R. C. (2015). gmodels: Various R programming tools for model fitting. R package version 2.16.2. Available online at: https://CRAN.R-project.org/package=gmodels.

[B32] Xue P. Sánchez-León E. Hu G. Lee C. W. Black B. Brisland A.. (2024). The interplay between electron transport chain function and iron regulatory factors influences melanin formation in *Cryptococcus neoformans* . Msphere 9 (5), e00250–e00224. doi: 10.1128/msphere.00250-24, PMID: 38687055 PMC11237718

[B33] Yin K. Wang Y. Zhao H. Wang D. Guo M. Mu M.. (2021). A comparative review of microplastics and nanoplastics: Toxicity hazards on digestive, reproductive and nervous system. Sci total Environ. 774, 145758. doi: 10.1016/j.scitotenv.2021.145758

[B34] Zhai Y. Guo W. Li D. Chen B. Xu X. Cao X.. (2024). Size-dependent influences of nanoplastics on microbial consortium differentially inhibiting 2, 4-dichlorophenol biodegradation. Water Res. 249, 121004. doi: 10.1016/j.watres.2023.121004, PMID: 38101052

[B35] Zheng Y. Ding W. Zhang T. Zhao Z. Wang R. Li Z.. (2021). Antimony-induced astrocyte activation *via* mitogen-activated protein kinase activation-dependent CREB phosphorylation. Toxicol. Lett. 352, 9–16. doi: 10.1016/j.toxlet.2021.09.006, PMID: 34571074

