# Supplementary material for: Polystyrene nanoparticles reduce the Cryptococcus neoformans virulence via induction of mitochondrial dysfunction
Source: Front Cell Infect Microbiol. 2025 Nov 5;15:1708192. doi: 10.3389/fcimb.2025.1708192 (PMC12626963; doi:10.3389/fcimb.2025.1708192)
Supplement: Supplementary Table 1 — Primer sequences used for qRT-PCR. F: forward primer; R: reverse primer. [file Table1.docx]

**TABLE S1** Primer sequences used for qRT-PCR. F: forward primer; R: reverse primer.

| Name | Sequence 5’-3’ | Reference |
| --- | --- | --- |
| Arg1_F | aagagtcagtgtggtgctgg | This Study |
| Arg1_R | tgtcagtgtgagcatccacc | This Study |
| Tnf_F | gatcggtccccaaagggatg | This Study |
| Tnf_R | ctacgacgtgggctacagg | This Study |
